# Identifying Factors Associated With Heightened Anxiety During Breast Cancer Diagnosis Through the Analysis of Social Media Data on Reddit: Mixed Methods Study

**DOI:** 10.2196/52551

**Published:** 2024-12-05

**Authors:** Joni Pierce, Mike Conway, Kathryn Grace, Jude Mikal

**Affiliations:** 1 Department of Biomedical Informatics University of Utah Salt Lake City, UT United States; 2 School of Computing & Information Systems University of Melbourne Melbourne, Victoria Australia; 3 Department of Geography, Environment, and Society University of Minnesota Minneapolis, MN United States; 4 Minnesota Population Center University of Minnesota Minneapolis, MN United States

**Keywords:** breast cancer, anxiety, NLP, natural language processing, mixed methods study, cancer diagnosis, social media apps, descriptive analysis, diagnostic progression, patient-centered care

## Abstract

**Background:**

More than 85% of patients report heightened levels of anxiety following breast cancer diagnosis. Anxiety may become amplified during the early stages of breast cancer diagnosis when ambiguity is high. High levels of anxiety can negatively impact patients by reducing their ability to function physically, make decisions, and adhere to treatment plans, with all these elements combined serving to diminish the quality of life.

**Objective:**

This study aimed to use individual social media posts about breast cancer experiences from Reddit (r/breastcancer) to understand the factors associated with breast cancer–related anxiety as individuals move from suspecting to confirming cancer diagnosis.

**Methods:**

We used a mixed method approach by combining natural language processing–based computational methods with descriptive analysis. Our team coded the entire corpus of 2170 unique posts from the r/breastcancer subreddit with respect to key variables, including whether the post was related to prediagnosis, diagnosis, or postdiagnosis concerns. We then used Linguistic Inquiry and Word Count (LIWC) to rank-order the codified posts as low, neutral, or high anxiety. High-anxiety posts were then retained for deep descriptive analysis to identify key themes relative to diagnostic progression.

**Results:**

After several iterations of data analysis and classification through both descriptive and computational methods, we identified a total of 448 high-anxiety posts across the 3 diagnostic categories. Our analyses revealed that individuals experience higher anxiety before a confirmed cancer diagnosis. Analysis of the high-anxiety posts revealed that the factors associated with anxiety differed depending on an individual’s stage in the diagnostic process. Prediagnosis anxiety was associated with physical symptoms, cancer-related risk factors, communication, and interpreting medical information. During the diagnosis period, high anxiety was associated with physical symptoms, cancer-related risk factors, communication, and difficulty navigating the health care system. Following diagnosis, high-anxiety posts generally discussed topics related to treatment options, physical symptoms, emotional distress, family, and financial issues.

**Conclusions:**

This study has practical, theoretical, and methodological implications for cancer research. Content analysis reveals several possible drivers of anxiety at each stage (prediagnosis, during diagnosis, and postdiagnosis) and provides key insights into how clinicians can help to alleviate anxiety at all stages of diagnosis. Findings provide insights into cancer-related anxiety as a process beginning before engagement with the health care system: when an individual first notices possible cancer symptoms. Uncertainty around physical symptoms and risk factors suggests the need for increased education and improved access to trained medical staff who can assist patients with questions and concerns during the diagnostic process. Assistance in understanding technical reports, scheduling, and patient-centric clinician behavior may pinpoint opportunities for improved communication between patients and providers.

## Introduction

### Background

In 2020, more than 2.3 million women worldwide were diagnosed with breast cancer, and over 685,000 died from the disease. Every 14 seconds, a woman is diagnosed with breast cancer worldwide, and in the United States, someone is diagnosed every 2 minutes. Breast cancer is the most common cancer diagnosis in 140 of 184 countries worldwide. In the United States, it is the most common cancer after nonmelanoma skin cancer [1,2]. Research has demonstrated that breast cancer diagnosis is associated with an increase in anxiety surrounding self-concept, mortality, cancer recurrence, treatment, and altered body image [3-7]. Furthermore, increased anxiety is associated with impaired physical functioning, reduced quality of life, decision-making ability, delayed return to work, and poor adherence to treatment [8]. Yet, for many, anxiety has its roots much earlier in the process of a cancer diagnosis, specifically when an individual first notices physical changes or risk factors that may represent an increased risk of cancer diagnosis, and for many, this increases stress [8,9]. Researchers have largely acknowledged increased anxiety following diagnosis [10,11], but less research has explored the anxiety associated with suspecting one may have breast cancer [12]. Untreated anxiety increases and amplifies the emotional and physical symptoms of patients with breast cancer. By increasing understanding of the relationship between anxiety and breast cancer, clinicians can provide more advanced interventional care to support better overall patient well-being. Advancing the scientific understanding of the ways that individuals experience anxiety during the different stages of cancer diagnosis, including the very early stages when breast cancer is suspected rather than officially diagnosed, provides an opportunity to support whole-person care. By connecting and cotreating breast cancer diagnostics and elevated anxiety, clinicians are better able to manage the process of adverse health diagnoses with mental health outcomes. Considering the ways these 2 health processes interact allows clinicians to provide appropriate support for an individual’s mental health as they proactively seek medical treatment.

Evaluating mental health outcomes like anxiety after an individual suspects disease but before they have received a diagnosis requires specific types of data capable of providing insight into each individual person’s dynamic mental and physical health status over a very specific time period related to the diagnosis. Time-varying, individual-level data capable of capturing the linkages between these processes are rarely, if ever, collected in studies of breast cancer and anxiety because it requires information about individuals based only on suspicion of disease rather than a medical diagnosis. The goal of this study is to explore anxiety, with specific attention to potential drivers of anxiety, across the diagnostic continuum during breast cancer diagnosis for people who are in different diagnostic phases. To conduct this research, we designed a unique dataset derived from social media posts, analyzed and interpreted through computational and descriptive methods. We collected data from the Reddit community r/breastcancer. Reddit is a social media platform that focuses on community engagement by offering subcommunities known as “subreddits” of specific areas of interest, like breast cancer. Reddit has over 1 billion registered users, with 47% of active users in the United States [13]. We analyzed all thread-initiating posts from the r/breastcancer subreddit. We began by assessing all posts to determine what stage in the diagnostic process the original poster (OP) is. We then used computational methods to identify posts characterized by a high relative frequency of anxiety-related terms. The “high-anxiety” posts were then retained for descriptive analysis to determine key themes that could provide insight into factors associated with anxiety and to assess whether those topics differ based on the individual’s stage in the diagnostic process.

### Theoretical Framing

Research has shown that up to 85% of patients with breast cancer experience elevated rates of anxiety related to changes in body image and sexual functioning, new responsibilities regarding treatment and treatment management, personal relationships, and logistical and financial concerns. Lazarus [14] defines stress as a multistage process beginning with assessment and ending with coping strategies. Opton and Lazarus [15] go on to describe the perception and interpretation of stress in several stages, beginning with an assessment of the stress event as an anticipation of harm. This event is then categorized as harmful, benign, or beneficial. According to Lazarus [14], cognitive appraisal of a threat is influenced by personal factors and situational factors. Personal factors include motivation, belief, intelligence resources, education, and knowledge. Situational factors include novelty, predictability, event uncertainty, temporal factors, and ambiguity [14,16]. When coupled with high levels of uncertainty, stress may be experienced as anxiety. Lazarus [14] defines ambiguity as a lack of situational clarity in contrast to uncertainty, which relates to a person’s confusion about the meaning of the environmental situation. Ambiguity can intensify a threat by limiting a person’s sense of control and increasing a sense of helplessness over the perceived danger. Monat et al [17] link anxiety to uncertainty about the nature of a threat, including the probability and timing of experiencing the threat, as well as an understanding of what can be done about the threat [17,18]. Hilton [19] describes coping resources, according to Folkman et al [20], to include planful problem-solving, confrontation, distancing, self-control, seeking social support, accepting responsibility, escape-avoidance, and positive reappraisal. Our study focuses on the coping strategy of support seeking by a specific breast cancer social media community hosted on the social media platform Reddit.

Anxiety levels can be categorized as “state anxiety,” meaning the anxiety is associated with a condition or situation, whereas “trait anxiety” is the propensity to worry and experience fear on a regular basis. Our study focuses on conditions of “state anxiety” related to the suspicion of having breast cancer. A total of 45% of patients reported severe state anxiety in the early stages of breast cancer diagnostics and treatment [9,21,22]. These heightened levels of anxiety can result in a host of adverse mental and physical health outcomes alongside diminished quality of life, potentially negatively impacting both immune response and cognitive functioning [23,24].

While nearly all patients with breast cancer experience some anxiety surrounding diagnosis, research has identified risk factors that have been shown to exacerbate anxiety among patients with breast cancer [4,25]. These risk factors can be divided into four distinct categories: (1) staging and cancer progression, (2) mental health history, (3) physical symptoms, and (4) patient (demographic) characteristics. Research on “staging and cancer progression” shows higher levels of anxiety for individuals diagnosed with metastatic breast cancer [25,26], while “mental health predictors” include a prediagnosis history of diagnosed anxiety or depression [3,25,27-30] or a precancer history of diagnosed personality disorder [27]. “Physical symptoms,” including pain, fatigue, insomnia, digestive disorders, and mobility issues, were associated with elevated anxiety levels during treatment and up to 12 months following treatment completion [5,10,31]. Patient or demographic characteristics, including age and race, were also shown to influence anxiety levels both directly and indirectly [28,31-35].

The preponderance of research on breast cancer–related anxiety has focused on anxiety following cancer diagnosis. This postdiagnosis focus encourages a unilateral conceptualization of anxiety. Specifically, unlike trait anxiety, state anxiety is a transitory emotional state that depends on a host of context-level factors [36]. These context-level factors change as an individual moves from suspecting to confirming breast cancer diagnosis. These changes are associated with disruption and ambiguity and lead to heightened levels of anxiety [37]. More to the point, evidence suggests that cancer-related anxiety does not emerge at the time of diagnosis [12]. For example, Lerman et al [38] showed spikes in cancer-related anxiety associated with abnormal and potentially problematic breast cancer screenings. Furthermore, research exists to support the notion that not only do cancer-related anxieties first emerge before cancer diagnosis, but those anxieties may diminish in the aftermath of a confirmed diagnosis [39]. Taken together, these studies suggest that anxiety has roots much earlier in the diagnostic process and evolves as patients encounter new challenges and gather new information.

### Breast Cancer–Related Anxiety and Social Media Support

We collected data from Reddit, a virtual space where many users find support through sharing their testimonials and asking for advice. Social support has been shown to be an effective tool to help people cope with anxiety. The transfer of advice, resources, and information in response to a stressor has been shown to reduce anxiety and to buffer against the deleterious effects of stress through a variety of mechanisms and in a variety of contexts (for a broader discussion, see, eg, [40-43]). Despite this, changes in support needs often coincide with moments of limited support availability [44]. In light of this, many individuals have turned to breast cancer support networks created and maintained online. Often associated with benefits similar to those of face-to-face social support exchange, computer-mediated social support, like that potentially offered through Reddit, offers notable advantages in the transmission of social support, including anonymity [45], improved congruence between the nature of support sought and support received [46], reduced communication barriers [47], and increased agency in support seeking [48].

Support seeking in the context of online communities provides unique data opportunities for using automated text processing methods to measure the relative levels of anxiety among individuals at different stages of their breast cancer diagnosis journey and to identify high anxiety posts for subsequent descriptive analysis. Combining descriptive and computational methods, this study reconstructs a diagnostic timeline to examine the evolution of anxiety beginning when an individual first suspects breast cancer. We then use descriptive analysis to identify factors associated with anxiety and how those factors shift before, during, and following a cancer diagnosis. Our mixed methods, computational-descriptive analysis is designed to identify possible factors associated with anxiety among individuals suspecting breast cancer and to observe how those factors evolve through the diagnostic and treatment processes.

## Methods

### Overview

The objective of this study was to identify potential contributors to high levels of anxiety among individuals suspecting breast cancer. Relying on Reddit’s broad and diverse user base, we extracted breast cancer and anxiety data from the r/breastcancer subreddit, a community for individuals who suspect or have been diagnosed with breast cancer (or are supporting someone who has).

We approached this objective using a mixed methods analysis consisting of three phases: (1) exploratory analysis for codification and categorization of cancer-related posts into broad categories; (2) computational analysis of linguistic markers of elevated anxiety and identification of original posts characterized by high levels of anxiety; and (3) descriptive analysis for the evaluation of statistical differences in anxiety levels between categories, analysis of original posts for both principal causes of anxiety, as well as the evolution of those anxieties over time [49-51].

**Figure 1:**
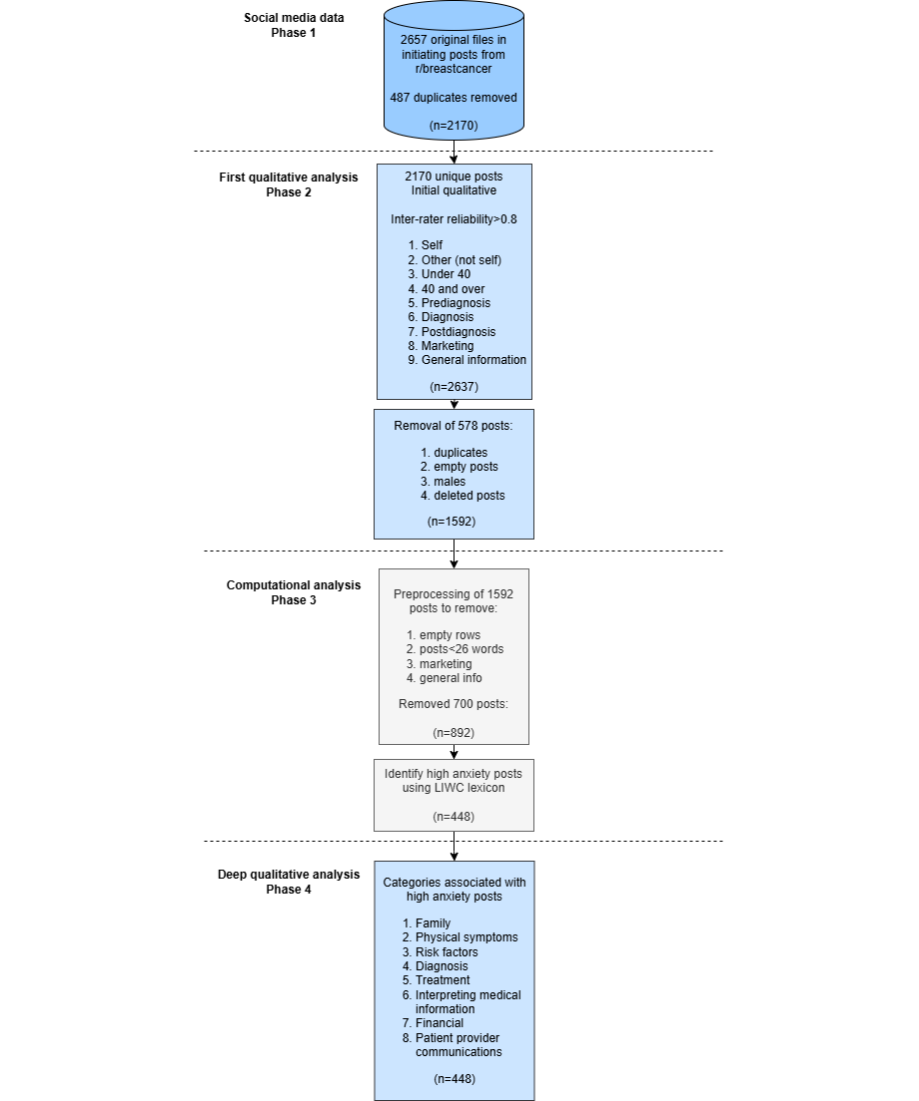
Multiphase analytic approach. IRR: interrater reliability; LIWC: Linguistic Inquiry and Word Count.

Our study focused only on high levels of “state anxiety” for different people who were in various diagnostic phases for breast cancer. State anxiety is defined as a transient state of arousal subjectively experienced as anxiety. It is a momentary emotional condition characterized by subjective feelings of apprehension and tension [21,22,52]. The analysis procedures and associated phases are depicted in Figure 1, and each phase is discussed in greater detail below (more details are provided in Multimedia Appendix 1).

### Exploratory Analysis and Classification

Given our interest in anxiety and its associated factors, we focused on initiating posts or posts that propose a topic or question to which users can respond. Using the PushShift application programming interface (API), we collected all initiating posts from Reddit’s r/breastcancer subreddit. This yielded a total dataset of 2170 posts. Authors JM, JP, and MC evaluated all posts independently following a bottom-up coding technique, identifying broad content classifications that had the potential to influence the degree and nature of anxiety expressed in the online posts. Authors then met weekly to consolidate and collapse categories.

Bottom-up inductive analysis revealed three dimensions with the potential to influence the degree and cause of individuals’ anxiety: (1) whether an individual had a confirmed breast cancer diagnosis, (2) the age of the user, and (3) whether an individual was posting about their own or another person’s cancer diagnosis. In addition, 2 subsequent categories also emerged with considerable overlap between them: general information posts and advertisements. General information posts were posts that included general information about breast cancer, cancer treatment, or risk factors; advertisements included any solicitation of cancer patients for products and services and participation in scientific studies. General information and advertisement posts were often generated by bots or individuals other than those suspecting breast cancer; thus, these posts were excluded from final tabulation and analysis. In all, 1592 posts were retained for analysis.

The final coding classification scheme had nine dichotomous categories: (1) identification of breast cancer risk (prediagnosis), (2) engagement with medical practitioners to confirm breast cancer diagnosis (diagnosis), (3) confirmed breast cancer diagnosis (postdiagnosis), (4) aged younger than 40 years (under 40), (5) aged 40 years and older (40 and over), (6) posting about own breast cancer (self), (7) posting about another’s breast cancer (other), (8) general information about breast cancer, and (9) advertisements. After removing all posts for general information and advertisements, we retained 7 final classification categories, as shown in Table 1.

**Table 1:** Initial classification of Reddit posts.

Category	Variable type	Description
Self	y/n^a^	OP^b^ discusses own cancer risk or diagnosis.
Other	y/n	OP discusses someone else’s cancer risk or diagnosis.
Under 40	y/n	OP reported age younger than 40 years.
40 and over	y/n	OP reported age 40 years and older or did not report age.
Prediagnosis	y/n	Post refers to events or concerns that occur before seeking medical attention.
Diagnosis	y/n	Post refers to concerns during or resulting from engagement with the medical system while seeking out a diagnosis.
Postdiagnosis	y/n	Post refers to events or concerns following formal diagnosis.

^a^y/n: yes/no.

^b^OP: original poster.

To quantify the agreement between coders, we used Cohen κ. After 3 rounds of annotation (coders MC, JP, and JM), we achieved an agreement score of 0.81. Cohen κ>0.7 is generally considered an acceptable agreement [53].

### Computational Analysis Using Natural Language Processing

The data were first classified into the coding categories described above (self, other, age, etc), and we excluded posts that were duplicates, empty posts, and deleted posts. Following this, we used an automated text analytics method to assign an anxiety score to all 1592 posts (ie, a score that indicates the extent to which a particular comment exhibited anxiety). To achieve this, we used lexicons derived from the Linguistic Inquiry and Word Count (LIWC) 2007 lexicon [54,55], a widely used resource [56-58] that automatically quantifies the presence of salient psychological categories from the text (eg, positive affect, negative affect, cognitive processes, perceptual processes, and swearing).

In the context of social media analysis, LIWC lexicons have been used extensively to study the emotional and cognitive consequences of various scenarios, including (1) romantic breakup [59], (2) studying expressions of loneliness [60], and most salient to our current research question, (3) to study emotional expression in cancer survivors [61]. A key feature of LIWC that makes it well suited to our goal is the fact that “anxiety” is among the 63 psychological dimensions encoded in LIWC. The “anxiety” category consists of 91 terms (eg, alarm*, asham*, and uneas*) that can be used to quantify the extent to which a given text exhibits anxiety. Note that the asterisk is a wildcard that allows the pattern to match relevant derivational and inflectional morphemes (eg, the pattern alarm* will match “alarm,” “alarmed,” and “alarming”).

We used the LIWC anxiety lexicon in conjunction with the Python text-processing library, the Natural Language Toolkit [62]. We used the Natural Language Toolkit to first tokenize each comment into its constituent word tokens, then (programmatically) counted the number of words in each post that matched a term in the LIWC anxiety lexicon. We excluded posts relating to marketing and general discussion of breast cancer issues, as in this research, we are primarily interested in the lived experience of Reddit users experiencing breast cancer. From our starting point of 1592 posts, these preprocessing steps led to a final dataset of 892 posts.

We calculated anxiety scores to distinguish high-anxiety posts from low-anxiety posts. Anxiety scores were determined by first calculating the normalized frequency of LIWC anxiety terms per post expressed as a percentage. This approach accounts for the fact that posts varied in length. Next, we calculated the median percentage across the 892 posts and then converted the percentage to an ordinal variable (ie, 0 [None]=no LIWC terms; 1 [Low anxiety]=percentage>0 but less than the median percentage; and 2 [High anxiety]=percentage≥median value). Table 2 shows frequency counts for each category.

**Table 2:** Ordinal values.

Breakdown of ordinal variables	Score	Count, n
No anxiety	0	336
Low anxiety	1	108
High anxiety	2	448

### Descriptive Analysis of High-Anxiety Post Themes

The computational analysis and codification described in step 2 began with the analysis of 1592 posts. We removed 578 posts due to duplicates, empty posts, and deleted posts, which yielded a subset of 892 posts. Out of those, 448 posts were categorized as high-anxiety posts across (1) diagnostic stage, (2) patient age, and (3) self versus other. To account for multiple categorizations for a single post, we created 3 separate datasets for prediagnosis concerns, concerns surrounding diagnosis and care engagement, and concerns that emerged after a confirmed breast cancer diagnosis.

Posts were again analyzed using a bottom-up, constant comparison approach. Authors JM and JP immersed themselves in the datasets to identify what social media data reveals about the sources of anxieties among individuals suspecting breast cancer, along with factors that may exacerbate cancer-related anxiety, and how those anxieties may evolve between an individual’s discovery of cancer risk, through testing to eventual diagnosis.

Authors JM and JP independently coded 50 posts from each of the 3 datasets and met weekly to identify emerging categorical themes with the goal of identifying the array of themes that captured the largest number of original posts across all 3 of the datasets. Once the authors had completed the original coding of the first 150 posts, we met to consolidate and collapse themes. The final list of categorical themes is presented in the *Results* section.

Once the final list of anxiety categories had been established, authors JM and JP divided the remaining 448 posts between them, and each researcher independently evaluated each post. Mentions of each of the anxiety types were tabulated to track the frequency of each within and between each of the diagnostic stages: prediagnosis, diagnosis, and postdiagnosis. The authors also conducted a close reading to identify factors that may exacerbate anxiety or how anxiety is likely to evolve over time. Results of the computational analysis, tabulation, and descriptive analyses are presented in text and table form in the *Results* section.

### Ethical Considerations

This study involved a secondary analysis of publicly available data posted on social media. All data used in this study were anonymized and aggregated. An ethics review was not sought due to the public nature of the data, along with the absence of identifying information for the person creating the social media post. Informed consent was not required as each participant voluntarily posted their data in a public forum on social media. No compensation was offered (more details are provided in Multimedia Appendix 2).

## Results

Our cross-sectional analysis of anxiety among individuals suspecting breast cancer as they move from suspecting to screening for and eventually confirming a breast cancer diagnosis consisted of a three-phase analysis plan: (1) classification, (2) computational analysis of linguistic markers, and (3) deep detailed descriptive analysis of anxiety in social media posts. Results are organized according to these methods.

### Classification

Our first-round classification of the entire r/breastcancer subreddit (ie, 1592 original initiating posts) yielded three principal classification categories: (1) an individual’s stage in the diagnostic process (ie, prediagnosis, diagnosis, or postdiagnosis), (2) an individual’s age (ie, 40 and over or under 40), and (3) whether the post related to the poster’s own cancer experience or that of a loved one. Tabulations can be found in Table 3.

**Table 3:** Principle classification categories.

Principle classification categories and subcategories			Posts, n
**Diagnostic stage**			
	Prediagnosis	235	
	Diagnosis	185	
	Postdiagnosis	352	
**Patient age**			
	Under 40	298	
	40 and over	594	
**Person**			
	Self	539	
	Other	353	

### Computational Analysis

Out of the 892 initiating posts that remained after the preprocessing steps in phase 3, shown in Figure 1, a total of 336 posts were classified as having no evidence of anxiety, 108 were classified as low anxiety, and the remaining 448 were classified as high anxiety. Posts varied in length with a mean number of words of 193 (SD 155; median 152, range 26-1766). Our analysis (more details are provided in Multimedia Appendix 1) revealed that anxiety was substantially higher for younger individuals, individuals posting about their own cancer diagnosis, and individuals in the prediagnosis and screening phases of cancer diagnosis. More detailed descriptive statistics are shown in Table 4.

**Table 4:** Descriptive statistics.

Category	Posts (n=892), n (%)	Median anxiety score	Most common anxiety terms
All	892 (100)	2 (high anxiety)	Worried, scared, risk, worry, and anxiety
Under 40	298 (33.4)	2 (high anxiety)	Worried, scared, risk, worry, and anxiety
40 and over	594 (66.6)	1 (low anxiety)	Worried, scared, risk, anxiety, and worry
Self	539 (60.4)	2 (high anxiety)	Worried, scared, risk, anxiety, and worry
Other	284 (31.8)	1 (low anxiety)	Worried, scared, worry, afraid, and fear
Prediagnosis	235 (26.3)	2 (high anxiety)	Scared, worried, risk, anxiety, and worried
Diagnosis	185 (20.7)	2 (high anxiety)	Scared, worried, worry, anxiety, and nervous
Postdiagnosis	352 (39.5)	1 (low anxiety)	Worried, scared, risk, anxiety, and worry

### Descriptive Analysis of High-Anxiety Post Themes

General inductive analysis revealed 9 themes associated with high-anxiety posts (Table 5). While research on cancer and anxiety has typically focused on the stresses faced by patients with breast cancer following diagnosis, our results provide compelling evidence that not only does anxiety have its roots much earlier in the diagnostic process, but that anxiety manifests differently based on whether an individual is first discovering breast cancer risk, engaging with the medical system, or have already received a formal breast cancer diagnosis. Our findings also suggest that those risks may change as individuals move from one diagnostic group to the next.

General inductive analysis revealed 9 key themes (more details are shown in Table 5). Themes centered around (1) family concerns, (2) physical symptoms, (3) risk factors, (4) diagnosis, (5) treatment, (6) interpreting medical information, (7) financial, (8) patient-provider communication, and (9) emotional distress.

**Table 5:** Categorical themes from high-anxiety posts.

Anxiety theme	Definition
Family	Refers to the impact of potential diagnosis on children or family, preparation for life after diagnosis or after cancer, communicating diagnosis, or health concerns
Physical symptoms	Physical symptoms concerns indicating cancer, cancer type, or gravity
Risk factors	Concerns regarding family history, lifestyle, genetic predisposition, or carcinogenic exposure
Diagnosis	Issues with diagnosis, missed diagnosis, persistent symptoms, or inconclusive initial test results
Treatment	Questions regarding treatment, treatment decisions, or side effects
Interpreting medical information	Anxiety resulting from online research and Google searches; difficulty in understanding or interpreting medical reports or lab reports; and interpreting clinician behavior
Financial	Concerns regarding insurance, treatment costs, or costs of tests; job-related issues
Patient-provider communication	Confusion or anxiety resulting from engagement with health care staff and clinicians
Emotional distress	Difficulty in managing emotions; fear that emotions were interfering with daily functioning or thinking clearly

Characteristics of the 3 phases (prediagnosis, diagnosis, postdiagnosis) are listed in Tables 6-8 below. The tables show a clear evolution of cancer concerns. Prediagnosis concerns across age categories (40 and over) tended to focus primarily on physical symptoms and risk factors. Notably, 65 (83%) of the 78 self and under-40 posts included mention of physical symptoms, while 44 (83%) of the 53 self and 40-and-over posts included mention of changes in physical symptoms. A substantial number of posts also focused on health care engagement. Health care engagement posts often included requests for information about how, when, and from whom to seek care for marked physical changes.

**Table 6:** Prediagnosis data.

Anxiety-related categories	Prediagnosis			
	Self (n=131)		Other (n=14)	
	Under 40 (n=78), n (%)	40 and over (n=53), n (%)	Under 40 (n=78), n (%)	40 and over (n=53), n (%)
Family	3 (4)	4 (8)	0	0
Physical symptoms	65 (83)	44 (83)	3	7
Risk factors	30 (38)	22 (42)	1	3
Diagnosis	2 (3)	3 (6)	2	5
Treatment	3 (4)	3 (6)	0	1
Interpreting medical information	7 (9)	4 (8)	1	4
Financial	2 (3)	3 (6)	0	0
Patient-provider communication	12 (15)	4 (8)	1	3
Emotional distress	5 (6)	3 (6)	2	0

**Table 7:** Diagnosis data.

Anxiety-related categories	Diagnosis			
	Self (n=92)		Other (n=12)	
	Under 40 (n=48), n (%)	40 and over (n=44), n (%)	Under 40 (n=4), n (%)	40 and over (n=8), n (%)
Family	6 (13)	0 (0)	0 (0)	0 (0)
Physical symptoms	42 (88)	29 (66)	4 (100)	6 (75)
Risk factors	20 (42)	7 (16)	2 (50)	1 (13)
Diagnosis	9 (19)	9 (20)	2 (50)	1 (13)
Treatment	1 (2)	1 (2)	1 (25)	2 (25)
Interpreting medical information	19 (40)	5 (11)	0 (0)	1 (13)
Financial	4 (8)	2 (5)	0 (0)	0 (0)
Patient-provider communication	15 (31)	16 (36)	0 (0)	0 (0)
Emotional distress	4 (8)	3 (7)	0 (0)	0 (0)

**Table 8:** Postdiagnosis data.

Anxiety-related categories	Postdiagnosis			
	Self (n=70)		Other (n=14)	
	Under 40 (n=29), n (%)	40 and over (n=41), n (%)	Under 40 (n=4), n (%)	40 and over (n=10), n (%)
Family	14 (48)	8 (20)	0 (0)	0 (0)
Physical symptoms	5 (17)	12 (29)	3 (75)	7 (70)
Risk factors	4 (14)	4 (10)	1 (25)	3 (30)
Diagnosis	3 (10)	6 (15)	2 (50)	5 (50)
Treatment	14 (48)	24 (59)	0 (0)	1 (10)
Interpreting medical information	3 (10)	2 (5)	1 (25)	4 (40)
Financial	1 (3)	3 (7)	0 (0)	0 (0)
Patient-provider communication	1 (3)	5 (12)	1 (25)	3 (30)
Emotional distress	4 (14)	8 (20)	2 (50)	0 (0)

Moving from Table 6 to Table 7, the incidence of physical symptoms and risk-related anxiety remains very high. However, a closer read of the post content reveals that physical symptoms or risk factor discussions tended to present physical symptoms and risk factors primarily as a backdrop to more specific questions about treatment, financial distress, or issues with the health care engagement process.

During the diagnostic process, individuals tended to report more anxieties stemming from either patient-provider communication or interpreting medical information. Anxiety stemming from patient-provider communication frequently resulted from individuals’ own attempts to reduce ambiguity by gleaning additional information from clinician behaviors. Posters noted specific instances where technicians interrupted screenings and left the room. Posters also reported being contacted by clinic staff to move up an appointment date. When clinicians did not provide additional information, posters often interpreted these behaviors as indicating a potential problem.

“Interpreting medical information” might include instances where individuals were unable to decipher complex medical reports or cases in which individuals took to the internet to research their own symptoms. In one instance, an adolescent posted that he needed help translating a medical report for an English language–learning parent who had been provided screening results in English.

There are several notable changes in content themes in the postdiagnosis table (Table 8). Following diagnosis, individuals were significantly less likely to report physical changes and risk factors, which were dominant themes in the prediagnosis and diagnosis tables. Rather, anxious posts about an individual’s own cancer tended to focus on issues related to treatment, most notably in the under-40 group. These individuals were often seeking advice from individuals who had been through cancer diagnosis and treatment and could provide insights related to cancer treatment, treatment side effects, or reconstructive surgery.

Another marked shift that occurred in the postdiagnosis table was a shift in the proportion of posts about one’s own cancer. In the prediagnosis and diagnosis datasets, most of the high-anxiety posts were from individuals suspecting they may have breast cancer. These were individuals who were, themselves, seeking a breast cancer diagnosis (14/145, 10% of prediagnosis posts and 12/104, 12% of diagnosis posts). The postdiagnosis group was characterized by a significantly larger proportion of high-anxiety posts originating from someone other than the person with breast cancer (85/155, 55% of posts).

## Discussion

### Principal Findings

Many patients experience the highest levels of anxiety during the early stages of investigating a possible breast cancer diagnosis before a confirmed diagnosis. When diagnostic uncertainty is high, some patients seek information and communication through social media channels and online patient education sites. Our study found 9 categories associated with elevated anxiety levels with suspected breast cancer. These categories include family, physical symptoms, risk factors, diagnosis, treatment, interpreting medical information, financial, patient and provider communications, and emotional distress. We found categories of concern shifted by age (under 40 and 40 and over) across the diagnostic stages. In the prediagnostic stage, both age categories showed physical symptoms and risk factors associated with high anxiety. For patients younger than 40 years of age, patient-provider communication gaps and interpreting medical information contributed to frustration and associated anxiety. In the diagnostic stage, both age categories showed physical symptoms, and patient-provider communications were associated with high anxiety. For patients younger than 40 years of age, risk factors and interpreting medical information were associated with high anxiety in the diagnostic stage. In the postdiagnostic stage, we found a marked shift by age category in the factors associated with anxiety. Patients younger than 40 years of age showed high anxiety associated with family, while patients aged 40 years and older showed high anxiety associated with physical symptoms, emotional distress, and breast cancer in others close to them. Both age categories showed high anxiety associated with treatment.

Our deeper analysis of the content also revealed high levels of anxiety associated with navigating and engaging the health care system and financial matters. Patients experience increasing anxiety associated with understanding and interpreting the early physical symptoms associated with breast cancer and the anticipated course of diagnostic events. These gaps included a lack of empathy and confusing or limited medical information. Social support structures appeared as a high priority for people experiencing possible breast cancer diagnosis for themselves and the people close to them.

Our descriptive content analysis and post tabulations provide key insights into how anxiety manifests in each of the diagnostic phases: prediagnosis, diagnosis, and postdiagnosis. Notably, our results indicate a need for additional public information regarding early breast cancer warning signs. Many of the high-anxiety prediagnosis posts were associated with changes in physical symptoms or identification of new family or behavioral risk factors. Nearly all users participated in the breast cancer subreddit community to assess their likelihood of a breast cancer diagnosis based on the physical symptoms and risk factors they identified in their original post. Several sought advice about whether the physical symptoms they described merited medical attention, cancer screenings, or where to seek treatment. Others who discovered risk factors were unclear about what should be done once they became aware of a high likelihood of a subsequent cancer diagnosis.

Results also pointed to the potential for improvements in patient-provider communication. For example, in addition to direct reports of feeling mistreated or dismissed by clinic staff, informational ambiguity emerged as a factor associated with elevated anxiety. High-anxiety posts were often related to difficulties understanding complex medical reports or understanding clinic or clinician behaviors (eg, leaving appointments midscreening to find an oncologist, scheduling follow-up appointments, or even moving previously scheduled appointments). Furthermore, many individuals used websites like WebMD or Google to try to understand medical reports, physical symptoms, risk factors, or the behavior of their clinician or clinical staff, but results of independent and unguided searches often contributed to individuals’ anxiety and ended up reported in patient posts as associated with anxiety.

Numerical and descriptive results are also highly suggestive that anxiety be viewed as a process and that the risk of high anxiety is actually higher before breast cancer is diagnosed. While we looked at a cross-section of data between individuals, our results show a clear evolution in the primary factors associated with anxiety based on an individual’s staging within the diagnostic process.

There was also a marked shift in the postdiagnosis table. In the prediagnosis and diagnosis groups, most of the original posters were individuals who were concerned about their own cancer. In the postdiagnosis table, over half of the posts came from individuals worried about the possible diagnosis of a close friend or family member. This shift may highlight the need for additional formal support structures for friends and family of cancer patients. The corresponding drop in the number of posts from patients with cancer may be an artifact of the more formal structures in place to provide support to individuals as they cope with treatment decisions, including social workers, nurses, and practitioners, as well as websites such as PatientsLikeMe.com. These structures and friendships may not be in place for individuals coping with the breast cancer of a loved one. The subreddit sites may provide invaluable secondary support. However, they may also signal the need for more formal networks of support and support groups for individuals providing care and support to a patient with cancer.

### Comparison to Previous Work

Our study findings are consistent with previous research results for studies focused on breast cancer and anxiety. These studies consistently report anxiety to be highest during the early stages of diagnosis when uncertainty is high, and the diagnosis may be undermined. Furthermore, uncertainty is associated with increased stress, which can translate to anxiety for some people [4,8-10,12,34,39,63-65]. We are not aware of other research studies that study the factors associated with high anxiety by age brackets across the diagnostic stages.

### Strengths and Limitations

By looking at individuals in different phases of the diagnostic process, we were able to obtain a good sense of what the evolution of stress might look like as individuals move from suspicion to confirmation of breast cancer. This approach may be advantageous in that our dataset includes the concerns of individuals who are ultimately not diagnosed with cancer.

First, our study does not include longitudinal data and, therefore, can only suggest a possible evolutionary trajectory associated with anxiety. The cross-sectional nature of the data does not allow for the study of self-reported experiences of state anxiety throughout the diagnostic phases for each patient.

Second, the data used in this study are not broadly representative of patients with breast cancer due to the source of the data, which is a social media forum. Contributors to health-related discussion platforms are estimated to represent only a small proportion of overall users. In a study of 63,990 social media users, van Mierlo [66] found that 90% of users were silent observers or “lurkers*”* rather than active participants [66]. In addition, 9% of users contributed sparingly, and only 1% were actively engaged in online dialogue. We estimate some derivative of these ratios translates to the r/breastcancer subreddit, thus limiting representation of the full range of views in this community.

Third, this study identified factors associated with elevated levels of anxiety related to suspected breast cancer. However, these associations do not provide evidence of causation for elevated anxiety. Consequently, the results of our findings may lead to inaccurate conclusions, including spurious correlations.

### Future Directions

Future studies could focus on longitudinal data associated with individual patients to understand the transient nature of state anxiety for individuals. Furthermore, experimental studies with informational interventions provided early in the diagnostic process could elucidate measurable effect data, which could guide future patient education and support tools aimed at reducing anxiety. More studies using new modalities, such as digital health interventions, could advance the research base for targeted and scalable patient information tools.

### Conclusions

The significance of this study is the identification of factors associated with high anxiety during the earliest stages of breast cancer diagnosis. The findings have been categorized by age and whether the expressed anxiety was related to self or another person (other). These categories can be used for individualized, targeted interventions to manage high-anxiety levels associated with breast cancer. Our findings in this study suggest that early intervention for anxiety during the breast cancer diagnostic process may help patients cope with high levels of anxiety found in the early and midstages of breast cancer diagnosis. Diagnostic delays and associated uncertainty appear to amplify breast cancer–related anxiety, indicating that communicating early and often is important. Access to medically sound information is critical since patients are using the internet and social communities to gather information and advice. While online communities offer immediate access to information, they can serve as a source of misinformation, which may exacerbate anxiety unnecessarily.

### Implications for Cancer Survivors

The findings of this study suggest that improving access and awareness around breast cancer information, peer coping communities, health coaching, and forward contingency planning would benefit patients who have high levels of anxiety related to breast cancer diagnosis. Focusing on person-centered care to include psychosocial support systems when people are coping with the possibility of breast cancer is expected to improve the anxiety associated with breast cancer investigation and diagnosis.

## Appendix

Multimedia Appendix 1 Descriptive and comparative statistics.

Multimedia Appendix 2 Institutional policy on ethics review.

## Abbreviations

API: application programming interface
LIWC: Linguistic Inquiry and Word Count
OP: original poster

## Data Availability

The datasets generated during and/or analyzed during this study are not publicly available to ensure the privacy of the Reddit breast cancer community members is maintained but are available from the corresponding author on reasonable request.
