# Supplementary material for: Identifying Factors Associated With Heightened Anxiety During Breast Cancer Diagnosis Through the Analysis of Social Media Data on Reddit: Mixed Methods Study
Source: JMIR Cancer. 2024 Dec 5;10:e52551. doi: 10.2196/52551 (PMC11659693; doi:10.2196/52551)
Supplement: Multimedia Appendix 1 [file cancer_v10i1e52551_app1.docx]

**Multimedia Appendix 1**

**Table S1. Statistical Analysis:** Range of word counts: 26 words to 1766 words, mean: 193.62; median: 152.

**Descriptive Statistics**

| **Category** | **Description** | **Median Anxiety Score** | **Mean Anxiety Score** |
| --- | --- | --- | --- |
| **Age** | Under 40 | 2.0 | 1.302 |
|  | 40 and over | 1.0 | 1.037 |
| **Self/Other** | Talking about themselves | 2.0 | 1.234 |
|  | Talking about someone else | 1.0 | 1.018 |
| **Diagnostic Stages** | Prediagnosis | 2.0 | 1.312 |
|  | Diagnosis | 2.0 | 1.297 |
|  | Postdiagnosis | 1.0 | 1.020 |
|  | Comparing prediagnosis and diagnosis in terms of anxiety score with the Wilcoxon rank sums test for ordinal values did not yield a p value > 0.05 (i.e. there is little evidence of a difference in anxiety levels between prediagnosis and diagnosis). | n/a | n/a |

**Table S2. Comparative Statistics.**

| **Category**  **Comparison** | **Description** | **Wilcoxon rank sums test**  **(Scipy Implementation)** | ***P* values** |
| --- | --- | --- | --- |
| **Age** | Comparing under 40 and 40 and over in terms of anxiety scores | 3.56 | *P* <.001 |
| **Role** | Comparing self and other in terms of anxiety scores | 2.846 | *P* <.001 |
| **Diagnostic Stage** | Comparing prediagnosis and diagnosis in terms of anxiety score | 0.360 | *P =*.718 |
|  | Comparing prediagnosis and post diagnosis in terms of anxiety score | 3.378 | *P* <.001 |
|  | Comparing diagnosis and postdiagnosis in terms of anxiety score | 2.974 | *P* =.002 |
