# Supplementary material for: Identifying Factors Associated With Heightened Anxiety During Breast Cancer Diagnosis Through the Analysis of Social Media Data on Reddit: Mixed Methods Study
Source: JMIR Cancer. 2024 Dec 5;10:e52551. doi: 10.2196/52551 (PMC11659693; doi:10.2196/52551)
Supplement: Multimedia Appendix 2 [file cancer_v10i1e52551_app2.pdf]

SECONDARY ANALYSIS OF PUBLIC USE DATASETS

Background

Secondary analysis of publicly available data is a common method of research. One of the main sources of such data is the federal government (e.g., Bureau of Labor Statistics). Increasingly, federal agencies require investigators to make data collected with assistance of federal funds publicly available. In addition, many professional organizations and journals are establishing the norm that investigators make data sets used for the production of published scholarly papers accessible in public use data files to encourage scholarly replication of research.

Publicly available is defined as data that that is accessible to the general public, not restricted to researchers. Public use datasets are prepared with the intent of making them available for public use. The data available to the public are not individually identifiable. In some cases, data may have both publicly available de-identified data and restricted use data. Restricted use data is not considered publicly available.

Description

The University of Utah IRB has determined that data in the datasets listed below has been stripped of identifiers and is publicly available. As a result, research using this data does not rise to the level of "human subjects research," under the federal Common Rule, 45 CFR Part 46 and University policies, and, therefore, does not require IRB review. See, SOP 401a: Definition of Research Involving Human Subjects.

If a project will use a de-identified dataset that is not listed below, investigators are invited to submit an application for a determination of non-human subjects in ERICA, the University of Utah's electronic submission system. Investigators may select "Create a New Study Application" in ERICA and then select "Request for Non-Human Subject Research Review". The short application will be reviewed by the IRB staff and may request additional information or will make a determination that the project does not meet the definition of human subjects research.

Research projects that merge more than one dataset in such a way that individuals may be identified are not covered by this policy, and require prior IRB approval. Such proposals may be eligible for exempt determination. Investigators may submit a new study application for review.

Some of the databases listed below have restricted-use datasets that include identifiers, and therefore, must be approved by the University of Utah IRB before the research begins. Restricted use data are not publicly available and investigators must submit a new study application for review.

Research projects involving analysis of secondary data from the following datasets/repositories will NOT require prior IRB approval:

Autism Brain Imaging Data Exchange (ABIDE)
Better Access to Data for Global Interdisciplinary Research (BADGIR)
Behavioral Risk Factor Surveillance System (BRFSS) *Public-use datasets only*
Bureau of Labor Statistics *Public-use data files only*
Demographic and Health Surveys (DHS)
Fragile Families & Child Wellbeing Study
Inter-University Consortium for Political and Social Research (ICPSR) *Public-use datasets only*
LIS Luxembourg Income Study Database and the Luxembourg Wealth Study Database

Please contact the IRB Office at (801) 581-3655 or irb@hsc.utah.edu for additional guidance.

Medical Expenditure Panel Survey (MEPS)
National Center for Education Statistics *Public-use datasets only*
National Center for Health Statistics *Public-use datasets only*<br>• National Health and Nutrition Examination Survey (NHANES)<br>• National Health Care Surveys (NHCS)<br>• National Health Interview Survey (NHIS)<br>• Vital Statistics System (NVSS)<br>• Longitudinal Studies of Aging (LSOA)<br>• National Immunization Survey (NIS)<br>• National Survey of Family Growth (NSFG)<br>• State and Local Area Integrated Telephone Survey (SLAITS)
National Election Studies
National Center for Statistics and Analysis (NCSA), of the National Highway Traffic Safety Administration
Panel Study of Income Dynamics (PSID) *Public-use datasets only*
Roper Center for Public Opinion Research
Surveillance, Epidemiology and End Results Program (SEER) Research Data
U.S. Bureau of the Census

Submitting a Public Use Dataset for Pre-approval
Public use datasets that may be considered for inclusion on the University of Utah list of pre-approved data sources are:

1. Public use datasets posted on the Internet that include a responsible use statement or other confidentiality agreement for authors to protect human subjects (for an example, see the ICPSR's responsible use statement)
2. Survey data distributed by University of Utah principal investigators who can certify that:
   a. the data collection procedures were approved by an IRB that satisfies the Common Rule criteria for an IRB, and
   b. the dataset and documentation as distributed do not contain information that could be used to identify individual research participants.

In order for a public use dataset to be considered for inclusion in the above list, an investigator must submit the following information on potentially eligible datasets to the University of Utah IRB office prior to conducting research:

1. Name of dataset.
2. URL of the dataset or other information on how to obtain the dataset.
3. Abstract (one-page maximum) describing the content of the dataset and its potential use.

Please contact the IRB Office at (801) 581-3655 or irb@hsc.utah.edu for additional guidance.
